# Research and Exploratory Analysis Driven—Time-data Visualization (read-tv) software

**DOI:** 10.1093/jamiaopen/ooab007

**Published:** 2021-03-01

**Authors:** John Del Gaizo, Ken R Catchpole, Alexander V Alekseyenko

**Affiliations:** Biomedical Informatics Center, Department of Public Health Sciences, Medical University of South Carolina, Charleston, South Carolina, 29425, USA; Department of Anesthesia and Perioperative Medicine, Medical University of South Carolina, Charleston, South Carolina, 29425, USA; Biomedical Informatics Center, Department of Public Health Sciences, Medical University of South Carolina, Charleston, South Carolina, 29425, USA; Department of Public Health Sciences, Medical University of South Carolina, Charleston, South Carolina, 29425, USA; Department of Oral Health Sciences, Medical University of South Carolina, Charleston, South Carolina, 29425, USA; Department of Healthcare Leadership and Management, Medical University of South Carolina, Charleston, South Carolina, 29425, USA

**Keywords:** longitudinal visualization, change-point analysis, change point analysis, changepoint analysis, forecasting, R, Shiny, surgical safety

## Abstract

**Motivation:**

Research & Exploratory Analysis Driven Time-data Visualization (*read-tv*) is an open source R Shiny application for visualizing irregularly and regularly spaced longitudinal data. *read-tv* provides unique filtering and changepoint analysis (CPA) features. The need for these analyses was motivated by research of surgical work-flow disruptions in operating room settings. Specifically, for the analysis of the causes and characteristics of periods of high disruption-rates, which are associated with adverse surgical outcomes.

**Materials and Methods:**

*read-tv* is a graphical application, and the main component of a package of the same name. *read-tv* generates and evaluates code to filter and visualize data. Users can view the visualization code from within the application, which facilitates reproducibility. The data input requirements are simple, a table with a time column with no missing values. The input can either be in the form of a file, or an in-memory dataframe– which is effective for rapid visualization during curation.

**Results:**

We used *read-tv* to automatically detect surgical disruption cascades. We found that the most common disruption type during a cascade was training, followed by equipment.

**Discussion:**

*read-tv* fills a need for visualization software of surgical disruptions and other longitudinal data. Every visualization is *reproducible*, the exact source code that *read-tv* executes to create a visualization is available from within the application. *read-tv* is *generalizable*, it can plot any tabular dataset given the simple requirements that there is a numeric, datetime, or datetime string column with no missing values. Finally, the tab-based architecture of *read-tv* is easily *extensible*, it is relatively simple to add new functionality by implementing a tab in the source code.

**Conclusion:**

*read-tv* enables quick identification of patterns through customizable longitudinal plots; faceting; CPA; and user-specified filters. The package is available on GitHub under an MIT license.

## BACKGROUND AND SIGNIFICANCE

Longitudinal modeling analyses are widely employed among biomedical disciplines. Common techniques include visualization, and algorithms for changepoint analysis (CPA). Interpretation of the results often involves collaboration between computational practitioners and domain experts. Although it is difficult for a research tool to simultaneously serve both user types; there is a need to facilitate collaborative interactions, and ultimately the quality of their research products.

The tool is more likely to be adopted by other academic labs if it (1) supports their domain-specific data; (2) is open source and transparent; (3) provides a graphical UI for non-technical (subject domain expert) users; and (4) is built in a widely-used programming language, such as R. Several open-source R tools provide intuitive interfaces and plotting capabilities: esquisse^1^, ggplotgui^2^, ggThemeAssist^3^, SLIDER^4^, and TumGrowth^5^. However, they do not provide CPA, or interpolation functions to regularize irregularly-spaced event data.

While data cleaning and curation are vital components of data science projects, we did not aim to implement a tool that performs these steps. There are already powerful R tidyverse^6^ packages that provide this functionality. However, we did desire a tool that is written in R, so that it can readily inter-operate with these packages; which enables rapid development and visualization as the data is curated. Therefore, the tool complements, but not substitutes, the role of a data scientist on a research project.

### Changepoint Analysis (CPA)

Changepoint Analysis (CPA) refers to a set of a methods and algorithms that find where in a sequence, often a time-series, that a change occurs. Specifically, CPA algorithms try to find where a summary statistic, θ (e.g. mean or variance), changes value. Most CPA algorithms assume that the data is regularly spaced in time. Since events typically occur at irregular time intervals, *read-tv* provides functionality to regularize event data.

We did not develop novel CPA techniques, but incorporated 4 algorithms from the R `changepoint` package^7^: **(1) AMOC** (at most one change)^8^, which uses a likelihood ratio test to find at most a single point where the statistic changes. **(2) BINSEG** (binary segmentation) finds multiple changepoints through hierarchical clustering^9^^,^^10^. **(3) SEGNEIGH** (Segment Neighborhoods) finds an exact solution. In comparison, BINSEG finds an approximate solution that may not be the global minimum^11^. SEGNEIGH employs dynamic programming to reduce computationally time, but it still scales quadratically with sequence length. **(4) PELT** (pruned exact linear time)^11^ employs optimal partitioning techniques^12^, and a pruning algorithm to filter changepoint candidates. Like SEGNEIGH, PELT has the advantage of finding an exact solution; but with relatively low computational costs that approach linearity with sequence length given certain data assumptions.^7^Killick et al^7^^,^^11^^,^^13^ provide informative descriptions of these CPA algorithms.

### Motivating Example: Surgical Flow Disruptions

*read-tv* was originally designed as a surgical safety research tool for analyzing surgical work-flow disruption (FD) sequences. There is compelling evidence that FD sequences are informative indicators of error causation^14–26^. In addition, the data collection is repeatable^27^, and the analyses are quantitative^28–31^. Joseph et al.^19^ identify the following adverse outcomes that increase with high FD rates: patient mortality^22^, procedure duration^23^^,^^24^, procedure errors^20^^,^^21^, OR member stress^25^, and perceived workload^26^. Additionally, a snowballing effect may occur, where a series of FDs leads to a high rate of subsequent disruptions, or cascades^32^^,^^33^. Collection of these data is via direct observation; which requires careful training of observers, and close attention to sampling and coding strategies^34^ to balance data quality with the cognitive demands of data collection. A set of analytical tools are needed to explore both the quality of the observational method, and a wide variety of contextual, process, and outcome effects for exploring and improving systems safety in surgical procedures. Doing so serves to enhance the overall reliability and utility of this data collection approach by encouraging a standardized data format for event recording, while facilitating powerful and flexible analyses.

Therefore, we developed *read-tv* to simultaneously show the relationships between time, surgical case, event (disruption) occurrence/rate, and event properties such as surgical phase and disruption type. The surgical-disruption data includes (1) timestamp; (2) disruption type (communication, coordination, patient factors, etc.)^35^, and (3) observer notes. Certain recent datasets also include a severity metric. The user can explore different CPA techniques to detect changes in FD rates, and hence detect cascades. Current FD analysis is often performed with spreadsheets, which leaves advanced visualization and CPA analyses largely out of reach.

However, we realized that this application can support a wide audience outside the domain of surgical safety. This generalizability is further described in later sections.

## OBJECTIVES

**Figure 1.**
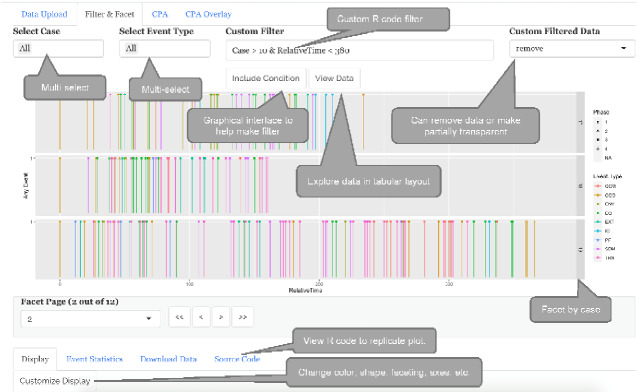
Flow Disruption by Time after Surgery Start, Faceted. *The filter and facet tab of read-tv supports faceting (Case), custom filters (Case > 10 and Relative Time < 380), different event colors (Event Type), shape (Phase), source code visualization, and other features shown above. This filtered and faceted data is passed to the CPA tab. The tabs to the right receive data from the tabs to the left.*

**Figure 2.**
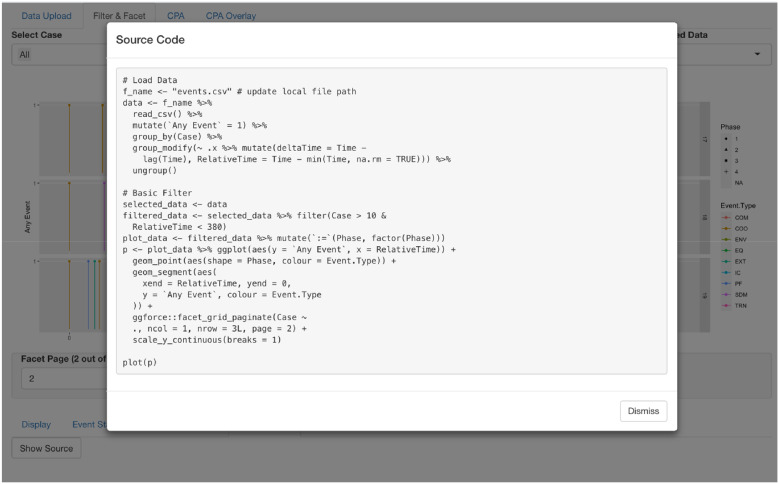
Source Code Generation. *Every read-tv visualization is the result of code generated through meta programming techniques in response to user input. The code that creates the plot can be visualized by pressing the “Show Source” button. The code above will recreate the plot in Figure 1.*

**Figure 3.**
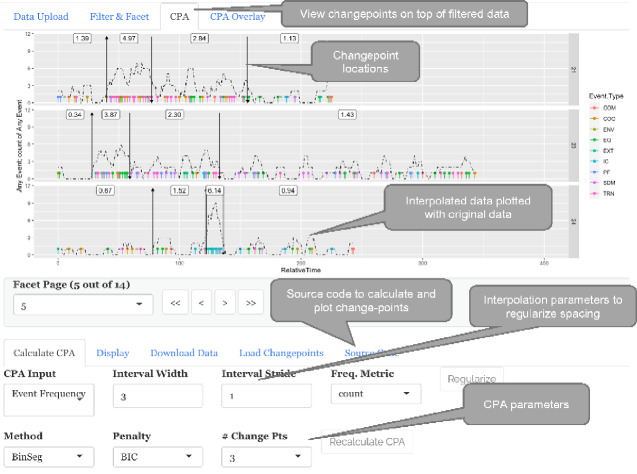
CPA Tab. *The CPA tab provides options to regularize timing intervals, prepare data for CPA input, and execute CPA algorithms. This is the same dataset as Figure 1, we just removed the custom filter in the “filter and facet tab” before calculating the changepoints*.

The main objectives for *read-tv* are largely portrayed in Figures 1–3, and are listed as follows.

- Web-support:
  - *read-tv* can be installed on a server so that is accessible to non-technical team members through the web interface.
- Event plots **(**Figure 1**)**:
  - For this category of longitudinal plots, the x-axis and y-axis correspond to time and event presence, respectively.
    - The y-axis is 1 if there was any event at the given time, otherwise 0.
    - *read-tv* creates a column, “Any Event”, which is simply 1 for each event and is plotted by default.
- Simple, tabular data input
  - Each row is an event or disruption, and each column is a property such as Time, Case, etc.
  - Supported file types are CSV, TSV, and RDS.
  - The user may pass in-memory dataframe structures from the R console if *read-tv* is on a local computer, which facilitates rapid analysis.
- Data filter **(**Figure 1**)**:
  - *read-tv* supports custom user-specified filters in R, which can also be generated through the GUI interface.
  - The filter should remove data from both the visualization and downstream analyses.
- Facet **(**Figure 1**)**:
  - We needed each surgical case to have a subplot.
  - Any column with 500 unique values or less can be used as a facet column.
- Facet pagination **(**Figure 1**)**:
  - This lets the user specify the number of subplots per page so that each subplot has sufficient space.
  - The pages can be scrolled.
- Color and shape **(**Figure 1**)**:
  - Events can be color or shape coded by properties such as disruption type.
  - Any column may be used for color coding.
  - Any column with 6 unique values or less can be used for shapes.
- Source code generation **(**Figure 2**)**
  - A user can view the R source code that *read-tv* generates to create an associated plot.
- Changepoint analyses (CPA)^36^**(**Figure 3**)**
  - Most events are irregularly spaced in time, i.e. inter-event time intervals are not consistent.
  - *read-tv* provides options to regularize the data, by counting the number of events at fixed intervals within a user-specified range. This can then be input into CPA algorithms.
  - *read-tv* enables the user to verify common CPA algorithms through output visualization.

## MATERIALS AND METHODS

### Design Approach

*read-tv* is a Shiny^37^ application that is distributed as a component of a package of the same. A how-to readme document and tutorial are publicly available: https://github.com/JDMusc/read-tv (last accessed January 04, 2021). It is developed as a package to facilitate installation and maintainable code organization.

The application is divided into tabs: Data Upload, Filter & Facet, CPA, and CPA Overlay (Figures 1–3). The tab outputs flow from left to right; in other words, the tabs on the left do not know about the tabs on the right. The CPA tab performs CPA on the output of the Filter & Facet. The CPA Overlay tab overlays the CPA output changepoints on top of the raw data from Data Upload. CPA Overlay also provides support for filtering and faceting data. However, the data filtered in CPA Overlay will not alter CPA tab’s changepoints (which are calculated from Filter & Facet’s output). This can be beneficial for seeing if certain event types are near changepoints, because event types not in question can be removed from the CPA Overlay tab’s visualization.

*read-tv*utilizes the changepoint^7^^,^^11^^,^^13^ R package and the tidyverse^6^ set of packages. These packages provide an infrastructure that supports *reproducible* visualizations, data input that is *generalizable* to diverse datasets, and an *extensible* graphic interface.

### Reproducibility

It can be challenging to document the steps to recreate a GUI-based visualization. Therefore, *read-tv* uses metaprogramming to generate code in response to user input. The code is evaluated to create visualizations. The user can view the code from *read-tv’*s “Source Code” option (Figure 1, 2). Therefore, any visualization can be *reproduced* by executing this code within an R environment.

### Generalizability

Though originally designed for surgical data, the application can be *generalized* for a wide range of longitudinal analyses through the principles of data simplicity and flexibility:

- Data simplicity:
  - *read-tv*ingests tabular data, with the only requirement that there is at least one column for time. Most data scientists know how to convert a given domain-specific model to this format.
  - Two optional data columns are Case and Event Type.
    - The Case column is used to calculate a relative time property, which is time since first event in the given case.
    - *read-*tv looks for Case and Event Type for default multi-select controls (Figure 1).
    - If these columns are absent, the user has an option to map them to other columns through a modal input.
  - If the column is neither provided nor mapped, then it’s multi-select will not be available (Figure 1).
  - If the Case column is neither provided nor mapped, then the relative time property will be calculated from the earliest point in the data.
- Data flexibility:
  - Time column:
    - The column for time does not need to be labeled as “Time”, the user can specify the column through a modal window.
    - The data format for the time column can be a datetime string, datetime (if the data is an R object), or numeric—such as number of minutes.
    - Most of the common string formats are supported as *read-tv* uses the tidyverse “readr” package to load data, which has functions that automatically detect most time formats.
- Flexible feature support:
  - As described in the objectives section, different columns can be used for faceting, filtering, color coding, or shape coding of events.

### Extensibility

*read-tv* has an *extensible* tab-based architecture. To add a custom tab to the application, a developer only has to modify 2 - 5 files in the original code base:

- *R/mainDisplayUI.R*
  - https://github.com/JDMusc/READ-TV/blob/master/R/mainDisplayUI.R (last accessed January 04, 2021)
  - The developer would add a line to instantiate and specify a name for the new tab.
- *R/mainDisplayServer.R*
  - https://github.com/JDMusc/READ-TV/blob/master/R/mainDisplayUI.R (last accessed January 04, 2021)
  - The developer would add a few lines to instantiate the tab’s logic, gain access to data and functionality from the previous tabs, and expose data or functionality that is output from the tab.

The following need to be modified to (1) import from another package, or (2) add sample data.

- *DESCRIPTION*
  - https://github.com/JDMusc/READ-TV/blob/master/DESCRIPTION (last accessed January 04, 2021)
  - This file lists dependencies and function imports.
- data folders
  - These folders contain sample data.
  - *data*
    - https://github.com/JDMusc/READ-TV/tree/master/data (last accessed January 04, 2021)
  - *data-raw*
    - https://github.com/JDMusc/READ-TV/tree/master/data-raw (last accessed January 04, 2021)

### Sample Data Sets

The read-tv package includes 3 sample data sets:

- *covid_usa*
  - NY Times data of daily new COVID-19 infections and deaths for each US state , 1/21/2020 - 9/21/2020^38^.
- *covid_global*
  - “Our World in Data” dataset of daily COVID-19 current cases, new cases, and deaths by country^39^.
- *japan_eq_3_11*
  - USGS provided seismic recordings from Japan during the Great Tohoku Earthquake in 2011^40^, ±1 day.

### Motivating Example

As mentioned in Background and Significance, the project’s motivating example was surgical disruption analysis. We used *read-tv* to develop a CPA model that quickly identifies surgeries where the disruption rate increases for a period of time, or a cascade. Figure 3 shows the CPA tab with specific CPA parameters. We defined a cascade as a period with an event rate of at least 1 event per minute over a 3-minute window (Figure 3). The cascade analysis source code can be found at: https://github.com/JDMusc/sugery-analysis (last acessed January 04, 2021).

Our dataset consisted of 41 RAS (robot assisted surgery) cases and 1,900+ disruptions. The RAS cases came from 4 sites and span a variety of surgical types. The FDs are categorized into 9 types, 7 from previous research^35^: (1) communication, (2) coordination, (3) equipment, (4) external factors, (5) instrument changes, (6) surgical decision making, (7) training: and 2 additional types: environment and patient factors.

## RESULTS

*read-tv* ingests relatively simple, tabular data where each row is an event. The data must have a time column without missing values. *read-tv* calculates 2 new columns: (1) Relative Time, the time since earliest event (within each case if a case column is provided), and (2) a column of all ones, Any Event. By default, *read-tv* produces simple stem plots of Any Event by Relative Time. However, the user can customize the x and y-axes, plot size, number of items per page for faceted plots, and map columns to colors or shapes. *read-tv* also supports data filtering through a graphical interface and with R queries (Figure 1). The Source Code sub-tab displays code to recreate the plot (Figure 2).

### Motivating Example

We found 25 cascades out of 41 surgeries; that 23 surgeries (56.1%) had 1 cascade, and that 1 surgery (2.4%) had 2 cascades; and that the training disruption type was the most common trigger of a cascade, followed by communication and equipment factors (7, 5, and 5 out of 25, respectively); the main FD type that is overrepresented during a cascade is training (Table 1). Training disruptions account for more than one-fourth of cascade disruptions (27%), 19% of general disruptions, and only 17% of non-cascade disruptions. As high disruption rates are correlated with negative outcomes, system changes that focus on decreasing training disruptions may have the highest impact on patient safety. As stated earlier, this analysis can be found at: https://github.com/JDMusc/surgery-analysis

**Table 1.** Cascade disruption types and patterns

Event Type	In-Cascade Count	Disruption Count	P(cascade|ET)	P(ET)	P(ET|cascade)	P(ET|notcascade)
TRN	117	376	0.31	0.19	0.27	0.17
COM	64	278	0.23	0.14	0.15	0.14
ENV	4	18	0.22	0.01	0.01	0.01
SDM	32	148	0.22	0.07	0.07	0.08
IC	26	126	0.21	0.06	0.06	0.06
EQ	90	460	0.20	0.23	0.21	0.24
PF	11	58	0.19	0.03	0.03	0.03
COO	71	385	0.18	0.20	0.17	0.20
EXT	13	125	0.10	0.06	0.03	0.07
**Any Event**	**428**	**1974**	**0.22**	**1.0**	**1.0**	**1.0**

ET stands for Event Type. The disruption type abbreviations are as follows: TRN, Training; COM, Communication; ENV, Environment; SDM, Surgical Decision Making; IC, Instrument Change; EQ, Equipment; PF, Patient Factors; COO, Coordination; EXT, External. The bottom row is bolded as the sum of above rows.

## DISCUSSION

Data visualization serves as an interdisciplinary communication bridge between quantitative scientists and domain experts. *read-tv* can (1) create *reproducible* visualizations through source code generation, (2) be *extended* through custom tab addition, and (3) be *generalized* to different datasets.

It was simple to both (1) heuristically set the CPA parameters for sample cases with the *read-tv* interface for exploring CPA plots, and (2) verify the validity of the CPA model simultaneously for many cases with faceted and paginated plots. Based on the literature reports that high disruption rates are associated with negative outcomes^20–26^, an algorithm that automatically detects and filters cases with high rates can help surgical safety researchers know which cases merit focus in order to identify system-level patterns that lead to error.

We ensured that *read-tv* was suitable for the exemplar purpose of analyzing flow disruptions in surgical care by developing it in close collaboration with the research team members who perform the observational data collection. The functional flexibility of the tool has substantially facilitated the range and speed with which analyses can be conducted. For example, we are in-process of publishing a separate inter-rater analysis. *read-tv* allows the research team to ascertain each individual event, faceted by rater. We can quickly compare the ratings (either severity or disruption type) for a series of disruptions, and heuristically inspect inter-rater reliability. This complements the more traditional approach of comparing summary statistics, by providing a granularity and rigor to disruption assessment that is usually unavailable.

## LIMITATIONS AND FUTURE WORK

The code was written by one person, under helpful guidance from mentors, and feedback from lab members. While the application was tested on surgical data, and the main developer tested it on some non-surgical data, we hope to receive feedback on GitHub as it is tested on a broad and diverse collection of datasets. We aim to turn *read-tv* into a community supported, open-source project.

The main future work is the incorporation of forecasting techniques into the application, which is currently under development.

## CONCLUSION

*read-tv* is an open-source, web application developed in R that provides features that fill a current research tool gap in longitudinal modeling. It has been positively received by surgical safety end users in our lab, and it can be applied to a wide array of use cases including general, urological, cardiac and cancer surgeries. As well as non-surgical medical data, and even general longitudinal data.

## Data Availability

There were four mentioned datasets, they are all accessible in public repositories. *covid_usa* NY Times data of daily new COVID-19 infections and deaths for each US state , 1/21/2020 - 9/21/2020^38^. https://raw.githubusercontent.com/nytimes/covid-19-data/master/us-states.csv (last accessed December 2020) *covid_global* “Our World in Data” dataset of daily COVID-19 current cases, new cases, and deaths by country^39^. https://ourworldindata.org/coronavirus-source-data (last accessed December 2020) *japan_eq_3_11* USGS provided seismic recordings from Japan during the Great Tohoku Earthquake in 2011^40^, ±1 day. https://earthquake.usgs.gov/fdsnws/event/1/query.csv?starttime=2011-03-10%2000:00:00&endtime=2011-03-12%2023:59:59&maxlatitude=45.919&minlatitude=26.861&maxlongitude=149.414&minlongitude=129.023&minmagnitude=2.5&orderby=time (last accessed December 2020) *PHI-Free Disruptions* ^41^ https://github.com/JDMusc/surgery-analysis/blob/master/data/events_phi_free.csv (last accessed February 04, 2021) DOI : 10.5061/dryad.d51c5b02g
